# Supplementary material for: Are carbon emissions trading and green financial instruments synergistic? -Comprehensive quantitative research based on content analysis
Source: PLoS One. 2024 Mar 7;19(3):e0298601. doi: 10.1371/journal.pone.0298601 (PMC10919601; doi:10.1371/journal.pone.0298601)
Supplement: S1 Data — (ZIP) [file pone.0298601.s001.zip › Data/地级市Z_F工作报告.zip!/╜¡╦╒/╬▐╬2╩╨/└┤╫╘╣1⁄2╓┌║┼í╛┬φ┐╦╩2╛▌═°í┐_2010.docx]

各位代表：

现在，我代表无锡市人民政府，向大会报告工作，请予审议，并请市政协委员和其他列席人员提出意见。

2009年工作回顾

2009年是新世纪以来我国经济社会发展最为困难的一年。一年来，在中共无锡市委领导下，无锡市政府以科学发展观为指导，紧紧依靠全市人民，积极应对国际金融危机，努力保持经济社会平稳较好发展。预计，实现地区生产总值5000亿元，同比增长11.5%左右，按常住人口计算人均地区生产总值达8.1万元；财政总收入1062亿元，增长16.8%，其中一般预算收入415.9亿元，增长13.8%；城镇居民人均可支配收入、农民人均纯收入均增长10%左右；城镇登记失业率2.8%；居民消费价格指数99.5，各项工作迈上了新的台阶。

（一）全力扩内需保增长，经济实现平稳较好发展

有效投入显著增长。积极扩大政府性投资，努力争取部省投资，狠抓一批重大基础设施项目、产业项目、环境和民生项目，全社会固定资产投资2387.6亿元，增长27.2%。落实中央投资项目市级配套资金31亿元，安排市本级经济发展专项资金7亿元。投资结构持续优化，工业技改投入增长16.7%，服务业投入占比达到55.3%。华润上华8英寸集成电路、尚德“冥王星”光伏电池等项目投产，海太半导体封装测试、中航无锡发动机控制工程中心、无锡粮食科技物流中心、宜兴油车水库等项目进展顺利。消费市场持续活跃。落实促进消费各项政策，实现社会消费品零售总额1651.4亿元，增长18.7%；百亿元市场增加到7家。全市商品房销售面积1007.4万平方米，增长87.5%。预计实现旅游总收入615亿元，增长18.2%。灵山景区成为国家5A级旅游景区。开放型经济逆势求进。积极加大政策扶持力度，努力调整出口产品结构和市场结构，强化出口品牌建设，大力培育新能源产品等新的出口增长点，进出口贸易出现向好势头。预计，进出口总额完成439.5亿美元，其中出口260.1亿美元。利用外资结构改善，高新技术和现代服务业外资占比大幅提升，成为产业结构调整的重要力量。预计完成到位注册外资32亿美元，增长1.1%。服务保障力度加大。积极开展银企合作，组织金融机构向1976户企业提供授信支持1640.5亿元。全市金融机构各项本外币存贷款余额分别达到7405.7亿元和5474.9亿元，其中中长期贷款占比提高到43.1%。实施增值税转型改革，执行企业所得税新政策，减免行政事业性收费，减轻企业税费负担。建成三级行政服务网络，全市6620项行政权力事项实现网上公开透明运行。通过向上争取和内涵挖潜，保障扩内需保增长项目用地3900多公顷。

（二）切实调结构促转型，经济竞争能力显著增强

三次产业结构优化。大力实施机械装备、纺织服装、现代商贸等十大产业调整提升计划，积极培育传感网、新能源、软件服务外包和文化创意等九大类重点新兴产业，预计高新技术产业增加值占规模以上工业增加值比重达到43.6%；服务业增加值占地区生产总值比重提高到42.5%；现代高效农业面积累计超过7万公顷，占全市耕地面积的50.6%。“科技创新和服务外包促进年”活动成效明显。设立市级创业投资引导资金10亿元，安排政产学研和国际科技合作专项资金1亿元。与“7+1”政产学研联盟院校签订合作项目累计达2500多项，研发投入超过75亿元。全市累计建成创新创业创意载体750多万平方米，集聚科技型企业2900多家。新增省级工程技术研究中心98家。预计全社会研发费用占地区生产总值比重达2.3%。无锡被列为中国服务外包示范城市。产业竞争能力增强。国家传感网创新示范区（国家传感信息中心）获国务院批准，中国物联网研究发展中心正式成立。国家火炬计划江阴风电装备产业基地、惠山风电关键零部件产业基地获批。中国邮政长三角速递物流集散中心落户无锡。兴澄特钢公司、无锡供电公司获无锡市长质量奖。新增中国驰名商标34件。16家企业集团入围2009中国企业五百强，入围企业数居全省首位。

（三）坚持统一规划统筹建设，城乡一体化进程明显加快

城市功能进一步提升。完善新城建设、老城改造和产业园区发展等重点规划，完成一批公共服务设施专项规划。太湖新城框架基本成型，锡东新城建设全面展开。惠山古街、清名桥等五大历史文化街区保护建设进展顺利，运河公园建成开放。城市管理和环境综合整治力度加大，主要街道包装出新，市容市貌得到改善。基础设施承载力进一步增强。地铁1号线正式开工。无锡综合交通枢纽、沪宁城际铁路惠山站和新区站加快建设。贡湖大道、新光路、红星路等城市道路投用，市区竣工通车城市道路215公里，其中市级组织实施101.5公里。无锡机场跑道加厚工程竣工，全年进出港旅客和货邮吞吐量分别达221.8万人次和4.7万吨。无锡江阴港迈入亿吨大港行列。锡苏高速、230省道宜兴段等对外交通工程完工，苏南运河市区景观段示范工程基本建成。500千伏江阴东等输变电工程竣工，川气东输入锡工程投用。太湖、长江“双水源”双向对置供水格局基本建立，中桥水厂深度处理、尾水处理和雪浪水厂尾水处理等工程完工。无锡建成国家节水型城市。新农村建设进一步加快。十大强农惠农工程全面完成年度任务，农村新五件实事总体目标提前实现。通过“两置换一转化”，新增耕地和农用地728公顷，完成土地整理5727公顷。新增农村公路300公里，改造农村危桥314座。新建为农服务社259家。村级经济股份合作社增加到446家，累计发放个人股红利近5亿元。在抓好无锡建设发展的同时，全力落实对口支援任务，其中援建四川汉旺地震灾区的卫生院、中心小学、幼儿园、自来水厂、新镇一期道路等公共设施和297套廉租房已交付使用。

（四）大力整治环境优化生态，生态文明建设扎实推进

太湖治理深入开展。加强饮用水源地保护，全面启动贡湖湾等湖岸湿地工程，沿太湖纵深200米范围内开展生态修复，建设防护林和入湖河道生态绿地。科学组织调水引流，完成太湖生态清淤197万立方米。加大蓝藻打捞力度，建成运行4座藻水分离站。建成污水管网700多公里，基本实现污水主管网全覆盖。全面推进控源截污“排水达标区”建设，全市68座污水处理厂全部达到一级A排放标准，污水日处理能力达到190万吨，全市城镇污水集中处理率达到85%。主要污染物入湖总量下降，太湖无锡水域水质改善，富营养化指数降至中度水平。节能减排完成良好。全市179个减排项目全部完成，预计主要污染物化学需氧量和二氧化硫排放总量在2005年基础上均累计削减30%。加强节能监督，严格项目能效评估，有效遏制高耗能项目建设。预计万元地区生产总值能耗下降4.5%以上。积极发展循环经济，45家企业完成强制性清洁生产审核验收。推行环境资源区域补偿和排污许可、有偿使用及排污权交易试点，集中开展环保专项行动，严查环境违法行为。绿色无锡建设成效凸显。全年造林绿化4680公顷，市区新增城市公共绿地837万平方米，人均公共绿地达13平方米，无锡成为省内惟一的“国家森林城市”。457个自然村完成村庄环境综合整治，全年建成绿色家园示范村147个、示范镇10个，新增国家级环境优美乡镇13个、国家级卫生镇8个。所辖宜兴市和锡山区、惠山区、滨湖区通过国家生态市（区）考核验收，新区创建成国家生态工业示范区，无锡市、江阴市被确定为国家生态文明建设试点城市。

（五）着力办实事惠民生，市民生活质量持续改善

就业形势稳中趋好。千方百计促进就业，降低社保费率，延长就业补贴期限，提高补贴标准，发放稳岗社保补贴1.1亿元，安排就业再就业资金4.1亿元，全市新增城镇就业10.8万人，扶持自主创业9577人，城镇失业人员再就业7.2万人。社会保障水平提高。全市企业养老、基本医疗、失业保险参保人数分别增加3.7万人、5.3万人、5.9万人，城乡养老保障覆盖总人数达341.9万人。41.9万名企业退休人员月人均增加养老金139.5元。新型农村合作医疗人均筹资水平超过300元，市区城镇居民医疗保险补助标准提高到98.5元。市区23家社区卫生服务中心按规范化标准建设到位，严格执行社区药品统一配送、零差率销售和社区卫生优惠及免费服务制度，药品价格和大型设备检查收费降低30%。市区及江阴最低生活保障制度实现城乡统一，其中市区月标准提高到380元。实事项目惠及群众。26项为民办实事项目全面完成。完成老新村整治和老住宅特修87万平方米，4558户家庭享受廉租住房保障。推进城乡公交一体化，实行统一票价，刷卡六折优惠，市区公交线路增加到201条，日客流量已超过100万人次。社区扁平化管理全面推开，累计建成城市社区事务工作站447家，农村社区服务中心603家。新增机构养老床位1800张、社区居家养老服务站82家，惠及老年人6万多名。加大对困难群众的慈善救助力度，支出慈善资金2.2亿元，为266名贫困家庭低龄残疾儿童提供免费康复训练，为1401户残疾人家庭进行无障碍设施改造。

（六）积极促和谐维稳定，社会发展水平有效提升

社会事业全面发展。坚持教育优先，全面实施义务教育学校教师绩效工资改革，义务教育优质均衡水平有效提升。加快教育布局调整，市辅仁高级中学、青山高级中学、聋哑学校及南菁中学、宜兴中学新校区全面建成，办学条件进一步改善。推进藕塘职教园区和各类职业院校建设，支持在锡高校发展提高，为无锡现代化建设提供人才支撑。公共文化服务体系建设和文化惠民工程取得新进展，无锡博物院科技馆等建成启用，农家书屋建设全面完成。阖闾城遗址入围中国十大考古新发现。积极推进对外友好交流，全市国际友城总数增加到30个。健康城市建设有力推进。建成4个省级全民健身工程和一批健身游园，无锡射击射箭馆投用。成功举办市第十届运动会，无锡籍运动员在十一届全运会上取得历史最好成绩。医药卫生体制改革全面启动。传染病、慢性病、职业病防治等公共卫生工作全面加强。市妇幼保健院新门诊大楼、精神残疾康复中心等建成投用。人口和计划生育工作取得新成绩，成为全国首批人口计生综合改革示范市。社会保持和谐稳定。畅通信访渠道，健全社会矛盾纠纷排查化解机制，切实维护群众合法权益。提升民族宗教事务服务管理水平，实现民族宗教领域团结和谐。严格加强食品药品和安全生产监管，全市食品安全检测合格率达96.8%，各类生产安全事故起数和死亡人数连续八年实现“双下降”，全市未发生重特大安全事故。健全社会治安防控体系，严厉打击违法犯罪行为，维护全市政治安定、社会安全、人民安宁的良好局面。史志、档案、侨务、对台、人武、人防、双拥、气象、防震减灾、无线电管理、应急管理等工作取得新成绩，妇女、儿童、老龄、残疾人等事业有了新发展。无锡市第二次全国经济普查任务圆满完成。去年3月，我市成功举办第二届世界佛教论坛，给海内外与会嘉宾和社会各界人士留下了深刻印象，赢得广泛赞誉，无锡的知名度、美誉度、影响力得到新提升。

2009年市政府以转变职能为核心，按照精简统一效能原则，全面实施机构改革，强化对决策和执行等环节的监督检查，加强廉政建设，推进权力阳光运行，确保各级行政管理机关依照法定权限和程序行使职权、履行职责。认真贯彻市人大及其常委会的各项决议，扎实办理人大代表的建议、批评、意见和政协委员的提案及建议案，努力提高政府行政执行能力和工作实效。

各位代表，2009年是新中国成立60周年。这一年，全市人民齐心协力，努力克服国际金融危机的严重影响，无锡经济社会发展保持良好势头，向率先基本实现现代化目标迈出了新的步伐。这些成绩来之不易，凝聚着全市人民的智慧和辛劳。在此，我代表无锡市人民政府，向在各个领域和不同岗位上辛勤劳动、无私奉献的全体市民，向给予政府工作积极支持的人大代表和政协委员，向各民主党派、工商联、各人民团体和各界人士，向中央及省在锡单位，向驻锡人民解放军、武警官兵和公安干警，向所有参与、支持和关心无锡建设和发展的海内外朋友，表示崇高的敬意和由衷的感谢！

各位代表，我们也清醒地看到，我市经济社会发展还面临不少困难和矛盾：经济持续增长的基础还不稳定、不牢固，社会投资和企业投资不足，消费需求对经济增长的贡献份额还不高，外部需求恢复困难很多；结构性矛盾仍较突出，传统产业技术水平和附加值偏低，新兴产业的发展壮大缺乏核心技术、领军人才和龙头企业，服务业的总量规模与发展水平都亟待提高。资源环境的瓶颈制约十分明显，生态环境质量还不稳定，资源节约型、环境友好型社会建设任重道远。城乡居民收入增长滞后于经济发展速度，社会保障层次还比较多、水平还不够高，城市管理、社会治安等方面还有一些突出问题亟待解决。此外，政府职能转变和作风建设仍有待进一步加强和改进。这些，都需要在今后工作中全力破解，重点突破。

2010年工作总体要求

进入新的一年，我市经济社会发展面临的形势依然复杂，积极变化和不利影响同时显现，短期问题和长期问题相互交织，内生因素和外部因素相互影响。当前，正处于国际金融危机影响减弱、世界经济有望恢复增长的转折期。国家宏观政策保持稳定，积极扩大内需、保持适度增长的措施扎实有力。从自身发展来看，无锡已进入工业化后期，这是一个产业优化升级、发展方式转变的重要拐点，也是一个创新发展模式、实现现代化跃升的关键时期。近年来，我市积极加大投入、力促转型，为后续发展积蓄了潜力和后劲；消费市场热点增多、日趋活跃，为经济增长提供了内源动力；城市现代化和城乡一体化进程加快，为无锡拓展了新的发展空间。我们必须科学把握后危机时代的形势变化，应对各种困难挑战，抢抓世界范围新技术革命和新产业发展机遇，全力争取赢得新的发展优势。

根据无锡所处经济社会发展阶段与实际情况，温家宝总理对无锡提出了建设“生态城、高科技产业城、旅游和现代服务城、宜居城”的殷切希望。“四城”定位，高屋建瓴，言简意赅，是对无锡现代化建设目标的高度概括和内涵提升，是无锡城市转型、发展方式转变与现代化建设的大目标和总方向。建设生态城，就是要把经济发展和环境保护有机统一起来，把建设“资源节约型、环境友好型”城市作为主要抓手，突出源头控制，强化综合治理，发展绿色经济，建设低碳城市，使无锡走上山川秀美、城市繁荣的和谐发展之路。建设高科技产业城，就是要集聚科教资源，吸引高端人才，全方位构筑以企业为主体、市场为导向的区域自主创新体系，全力推进科技成果产业化，培育区域高科技产业体系、企业体系、产品体系和品牌体系，构筑创新型产业高地。建设旅游和现代服务城，就是要把旅游业发展作为优化服务经济结构、完善城市功能、提升城市品牌的重要手段，使旅游业成为现代服务业的龙头、城市经济的战略性支柱产业；同时，围绕旅游核心功能，发展大旅游，带动大服务，促进现代服务业壮大规模、提高水平，全面提升中心城市发展能级。建设宜居城，就是要把市民福祉提高、生活环境改善和经济增长、城市功能完善结合起来，充分整合无锡的自然优势、经济优势和人文优势，全面系统地提升城市宜居品质，把无锡打造成一个市民自豪、生活舒适的现代化城市。

今年是实现“十一五”发展目标、谋划“十二五”规划的关键之年，也是全面启动“四城”建设的开局之年。做好今年工作，具有承前启后的历史意义。政府工作的总体要求是：全面贯彻落实中央和省的决策部署，以率先基本实现现代化为主线，以建设“生态城、高科技产业城、旅游和现代服务城、宜居城”为目标，按照“促转型、扩内需、稳增长、惠民生”的要求，把扩大内需作为基本立足点，把推动发展方式转变和经济结构调整作为主要着力点，把保障和改善民生作为根本出发点和归宿点，努力实现经济社会又好又快发展。

综合考虑各方面因素，今年经济社会发展的主要预期目标是：地区生产总值增长12%左右；万元地区生产总值能耗降低4%；主要污染物排放量在2005年基础上累计削减32%；环境质量综合指数达到90；地方财政一般预算收入同口径增长12%；城镇居民人均可支配收入和农民人均纯收入均增长10 %以上；城镇登记失业率控制在3.6%以内；居民消费价格指数控制在省定范围以内。

新的形势和任务，对政府工作提出了更高要求。工作中着重把握好以下几点：深化市场改革，完善政府调控。遵循市场经济规律，不断加强和改善政府经济调节能力，坚持不懈地推进改革开放，为经济社会发展提供强大动力和制度保障。着力扩大内需，努力稳定外需。坚持扩大内部需求特别是消费需求的方针，积极拓展外需市场领域和层次，实现外需与内需有效互补，着力提高经济国际竞争力。力推结构调整，加速城市转型。以“四城”目标定位引导经济结构、城乡结构和社会结构大调整，切实转变发展方式，全面提高区域竞争力和可持续发展能力，建设怡人宜居繁荣的现代化城市。坚持改善民生，增进社会和谐。更加注重围绕保障和改善民生来谋划发展，完善公共服务和社会保障，加强社会管理，维护安定团结的社会政治环境，促进人的全面发展和社会全面进步。

2010年主要工作任务

（一）提高驾驭市场经济能力，保持国民经济平稳较快发展

贯彻落实中央宏观调控各项政策措施，加强和改善经济调节与市场监管职能，巩固和增强经济回升向好势头。突出财政政策实施重点。加大对民生领域、社会事业、科技创新、生态环境、基础设施的保障力度，重点支持新兴产业发展。加强税收和非税收入征缴管理，继续从严控制一般性支出。创新政府投资项目建设管理运作机制，维持政府投资适度规模，重点用于在建项目。加强财政资金绩效审计，提高资金使用效率。鼓励民间投资。更加注重调动社会投资积极性，合理引导社会投资方向，拓宽社会投资领域。优化信贷结构，增加有效信贷投放，保障重点项目建设发展。鼓励金融机构开发适合中小企业特点的金融产品，鼓励社会力量兴办担保机构，鼓励发展风险投资和创业投资，不断拓宽融资渠道。全力推进和落实170个市级重点项目。全社会固定资产投资增长20%。扩大消费需求。完善收入分配制度和社会保障体系，着力提高居民经营性及财产性收入，努力扩大中等收入群体，加大对低收入群体的帮扶力度，稳定并提升消费预期。优化商业网点空间布局和业态结构，增强城市社区服务功能，健全农村流通网络，实现为农服务社全覆盖。适应群众生活多样化、个性化需要，培育消费热点，引导消费升级。强化市场信用建设，狠抓商品安全监管，构建食品安全保障体系。社会消费品零售总额增长18%。促进房地产业健康发展。完善住房保障体系，市区新开工建设保障性住房7500套，新增廉租住房700套。完成老新村整治改造和老住宅特修80万平方米，基本完成中心城区重点成片危旧住房和快速内环以内城中村改造任务。增加普通商品房用地供应，加快中低价位、中小套型普通商品住房建设，合理引导住房消费。继续加强市场监管，整顿和规范房地产市场秩序。

（二）加大经济结构调整力度，构筑现代产业体系

坚持以结构调整为主线，全面实施“新兴产业培育年”和“无锡千人计划促进年”活动，加快建设创新型经济领军城市和科技创业家摇篮城市，提高产业集聚资源、综合发展的能力。抢占产业竞争制高点。对电子信息、新能源、生物医药、新材料、软件和服务外包等重点发展的新兴产业，要聚焦项目，注重实效，培育具有国际竞争力的企业集团和企业家团队，建设一批特色产业基地。重点打造国家微电子产业基地，加快壮大集成电路、液晶显示、汽车总成及零部件等产业集群，着力发展光伏、风电装备等产业。尤其要把建设国家传感网创新示范区作为重中之重，高起点高标准编制发展规划，加快推进研发机构、标准体系、检测平台和应用示范工程建设，培育壮大一批专业企业，拓展传感产品应用市场。高新技术产业增加值占规模以上工业增加值比重提高到45%。全力创建国家创新型城市和国家知识产权示范城市，进一步拓展政产学研联盟，支持重大科技成果在锡实现转化、应用和量产。全社会研发费用占地区生产总值比重达到2.5%。提高产业附加值。加速运用高新技术改造提升机械、轻纺等传统优势产业，促进产业链向研发、设计、营销、服务等全过程延伸，推动支柱产业从数量规模型向品牌效益型转变。推广卓越绩效管理，鼓励企业主导和参与制订国际标准、国家标准和行业标准，推进名牌战略，建设国家商标战略实施示范城市。积极发展现代高效农业，加强农产品质量和品牌建设，加快农业标准化步伐，做大做强一批农业产业化龙头企业和现代农业园区，不断提高农业附加值。提升服务业发展水平。大力发展现代物流、金融、文化创意等新兴服务业，优化升级商贸流通、社区服务等传统服务业，服务业占比提高到43.5%。着力发展总部经济，推进企业主辅业务分离，加快现代服务业集聚区建设。要聚焦旅游业，创新旅游资源管理体制与旅游产业运行机制，大力培育发展旅游专业化企业，鼓励民间资本、外商资本开发旅游资源，实现旅游资源专业化、集约化、规模化经营。抢抓上海“世博会”契机，加快推进八大博览园等旅游新品建设，打造精品景区和精品线路，推动文化游、农业游、水上游等业态发展。加强旅游产品整体策划和创新包装，强化无锡旅游休闲目的地形象推广。

（三）突出生态城宜居城目标，打造现代化区域中心城市

坚持区域城市化发展导向，以城乡一体化为目标，统筹城乡发展，增强中心城市集聚辐射功能，致力打造个性鲜明、特色彰显的现代化山水湖滨城市。优化城乡空间布局。努力提高太湖新城公共服务配套能力，加快建设市民中心、会展中心和大剧院等重点工程，营造新城风貌特色。进一步完善锡东新城规划体系，全面推进启动区建设，力争建成路网主骨架。提升蠡湖新城品质，实施蠡湖水环境深度治理和生态系统修复，积极稳妥推进功能开发。有序推进城市更新，重点提升城市功能，完善配套设施和生态系统，做强做大中心城区。加强历史文化遗址保护，推进古街、古村、古镇保护建设，积极参与大运河申遗工作，加快创建国家历史文化名城群。严格保护耕地，依法集约节约利用土地，推进万顷良田建设和“两置换一转化”工作，完成土地整理4000公顷。强化重大基础设施建设。全面推进地铁1号线建设，力争启动2号线。加快推进京沪高速铁路和宁杭城际铁路无锡段及其站区配套设施建设，确保沪宁城际铁路无锡段如期建成。继续加大机场功能建设和航线开辟力度，进出港旅客人数达到260万人次，货邮吞吐量达到6万吨。完善城乡道路体系，建成环太湖高速公路硕放互通和锡张高速公路，推进高浪路东延、金城东路快速化改造等城市重点道桥项目。市区新建拓建城市道路100公里，全市建成农村公路200公里，改造农村危桥200座。改善生态环境质量。编制实施低碳城市建设规划，创建国家生态城市群，建设生态文明先驱城市。调整资源利用结构，推进能源计量工作，鼓励开发新能源，使用清洁能源，建设资源再回收利用体系。切实加强控源截污，推进入湖河道和城乡河塘整治，加快走马塘拓浚延伸工程建设，稳步推进梁塘河等八大湿地工程，完成太湖生态清淤426万立方米，主城区生活污水集中处理率提高到95%以上，实现主要污染物入湖总量继续下降，确保水环境质量持续改善。基本完成环城古运河风貌带综合整治。全年新增造林绿化面积4300公顷，市区新增城市绿地500万平方米。强化大气环境综合整治，扩大烟尘控制区范围，加强建筑工地综合管理。完善生活垃圾收运体系，市区建成区生活垃圾机械化收集率达95%。

（四）深化改革扩大开放，增强转型发展动力

围绕形成有利于科学发展的体制机制，不断深化改革创新，充分发挥对外开放的带动作用，进一步激发经济社会发展的动能与活力。优化所有制结构。完善市场竞争机制，放宽市场准入，激励民间投资，增强民营经济竞争力。鼓励企业改善经营机制，充分利用国内外资本市场，促进民营企业发展壮大。高度重视中小企业发展，推动中小企业技术进步和结构调整。深化国有资产管理体制改革，规范投资行为，完善监管制度，确保国有资产保值增值。深化重点领域改革。推进财税管理体制改革，强化政府资源性收益管理。探索建立综合性公共资源交易中心，形成统一市场，实行统一监管。强化三级行政服务体系功能，进一步理顺规范行政审批权限和事项，清理取消不合理收费项目。加快推进“资源节约型、环境友好型社会”综合配套改革试点工作。继续深化农村改革，壮大农村合作组织，加强土地承包经营权流转管理和服务，探索建立城乡统一的建设用地市场。提升开放型经济国际竞争力。开放型经济是无锡的优势和特色，任何情况下都不能懈怠和放弃。要创新理念，突出重点，增创对外开放新优势。实施多元化利用外资战略，继续加大招商引资力度，促进引资与引智相结合，引智与培育新兴产业相结合，着力引进有利于推动科技创新、产业升级和区域协调发展的外资项目。强化开发区资源优化整合，提升开发区功能，加快各类创新要素向开发区集聚。积极应对国际市场变化，进一步加强服务和引导，加大国际市场开拓、进出口信用保险等资金扶持力度，深化出口基地和品牌建设，促进外贸转型升级。进出口总额增长5%。完善信息服务网络，积极发展对外投资和经济合作，提高“走出去”成效，拓展经济发展空间。

（五）着力保障和改善民生，促进社会和谐进步

坚持以人为本发展理念，继续加大民生领域和社会建设投入力度，全力办好30项实事项目，稳步提高人民生活水平，促进社会全面进步。提升就业保障水平。继续实施积极就业政策，实现城乡就业优惠扶持政策全覆盖，重点援助帮扶大中专毕业生、农民工、就业困难人员等群体。新增城镇就业10万人，扶持自主创业3000人。扩大企业工资集体协商制度覆盖面，维护劳动者合法权益。探索建立城乡一体的居民养老保险和医疗保险制度，完成新型农村合作医疗与城镇居民医疗保险制度的整合并轨。进一步提高企业退休人员基本养老金，全市企业养老、基本医疗保险覆盖人数分别增加10万人和4万人。完善社会救助体系，提高社会救助覆盖面。提升公共服务能力。健全教育投入保障机制，全面实施素质教育，提高区域教育现代化水平。改造义务教育相对薄弱学校及办学点60所，完成城市职业技术学院、高等师范学校新校区建设。深化医药卫生体制改革，健全基层医疗卫生服务体系，全面推行基本药物制度，稳步推进公立医院改革试点，努力提升医疗服务水平。社区卫生服务中心探索实行收支两条线管理。完成市二院综合病房大楼建设，推进市中医院易地新建、三院改扩建、人民医院二期和食品药品检验检测中心等工程建设。建立健全分级投入机制，实施区域公交资源整合，进一步优化公交线网结构，增加公交运力，完善场站基础设施，全面完成公交智能化建设，公交分担率提高到25%。提升城市文明程度。深入开展群众性精神文明创建活动，争创全国文明城市。繁荣哲学社会科学，积极发展文学艺术、新闻出版和广播电视事业，加强网络文化建设与管理。提高免费开放公共文化设施服务水平。大力发展文化产业，完善产业链，培育骨干文化企业。全面实施《全民健身条例》，推进体育基本现代化试点工程，加强公共体育设施建设，完成体育公园改造扩建工程。积极备战十七届省运会。完善人口计划生育优质服务体系，夯实基层人口服务管理基础，全面提升人口素质。做好无锡市第六次全国人口普查工作。加强民族宗教工作。争创全国双拥模范城“六连冠”。继续结交国际友城，深化各领域国际交流与合作。提升社会管理效能。完善城管体制机制，落实各级管理职责，提高城管科技水平，实现市容市貌明显改善。深入推进社会矛盾化解、社会管理创新、公正廉洁执法三项重点工作。完善信访工作制度，扎实开展人民调解工作。进一步落实企业安全生产主体责任，确保安全生产形势稳定。严厉打击各类违法犯罪活动，深化社会治安防控体系建设，加强基层基础工作，提高平安创建的层次和水平，促进社会和谐稳定。

（六）强化政府自身建设，全面履行政府职能

围绕建设服务型政府，切实加强政府自身建设，转变工作作风，求真务实，埋头苦干，少说多做，更好地为发展服务、为基层服务、为群众服务。加快职能转变。全面完成市、市（县）区和乡镇政府机构改革任务，深化事业单位改革，理顺职责关系，优化机构设置，提高管理效能。健全应急管理体系，提高预防和处置突发公共事件能力。加强调查研究，健全民主集中、专家咨询、社会公示与听证等制度。编制好“十二五”国民经济和社会发展规划。严格依法行政。依照法定权限和程序行使权力、履行职责，依法管理经济社会事务，规范政府行政行为。全面落实市政府常务会议法律学习制度。继续推进政务公开，健全政府信息公开和新闻发布制度，提高政府工作透明度和公信力。自觉接受人大法律监督、工作监督和政协民主监督，主动接受群众监督和舆论监督。加强从严治政。弘扬务实作风，狠抓工作落实。加强重点工作、重大决策的跟踪督查，深入开展执行力监督检查，确保政令畅通。健全责任追究体系，严格实施领导干部问责制。全面落实廉政建设责任制，扎实推进无锡特色惩治和预防腐败体系建设，加强对行政权力运行的监督和制约，强化公务员队伍教育、培训和管理，努力建设高效廉洁的人民满意政府。

各位代表，新的形势催人奋进，新的征程任重道远。让我们紧密团结在以胡锦涛同志为总书记的党中央周围，在省委、省政府和市委的正确领导下，解放思想，开拓创新，团结拼搏，锐意进取，为率先基本实现现代化而努力奋斗！
